# Morphological and Molecular Identification of Peach Brown Rot Disease in Tibet and Exploration of the Biocontrol Efficiency of *Trichoderma*

**DOI:** 10.3390/jof8111174

**Published:** 2022-11-08

**Authors:** Shuwu Zhang, Dong Xiang, Chenxi Sun, Kaidi Han, Tong Li, Jingjiang Zhou, Bingliang Xu

**Affiliations:** 1 College of Plant Protection, Gansu Agricultural University/Biocontrol Engineering Laboratory of Crop Diseases and Pests of Gansu Province/State Key Laboratory of Aridland Crop Science, Gansu Agricultural University, Lanzhou 730070, China; 2 Institute of Vegetable, Tibet Academy of Agricultural and Animal Husbandry Sciences, Lhasa 850032, China; 3 State Key Laboratory of Green Pesticide and Agricultural Bioengineering, Ministry of Education, Guizhou University, Guiyang 550025, China

**Keywords:** biocontrol, brown rot, *Monilinia* species, morphological characterization and molecular identification, *Prunus persica* (L.) Batsch, Tibet, *Trichoderma* spp.

## Abstract

Brown rot caused by the pathogen of the genus *Monilinia* is the most destructive disease in peaches worldwide. It has seriously reduced the economic value of the peach (*Prunus persica* (L.) Batsch) in Nyingchi and Qamdo, Tibet, China. *Monilinia fructicola*, *Monilia mumecola,* and *M. yunnanensis* have been reported as the causal agents of brown rot disease on stone fruits in China. In this study, we report on the identification of *M. yunnanensis* in peach orchards in Nyingchi and Qamdo, Tibet. From twenty-three isolates with the same characteristics, we identified the representative single-spore isolates T8-1, T8-8, and T8-20 as *M. yunnanensis* and confirmed that the Tibet brown rot disease was caused by *M. yunnanensis* based on the morphological characteristics and molecular analyses. The phylogenetic analysis of the glyceraldehyde-3-phosphate dehydrogenase (*G3PDH*) and β-tubulin (*TUB2*) nucleotide sequences and the multiplex PCR identification revealed that the representative isolates T8-1, T8-8, and T8-20 were more closely related to *M. yunnanensis* than other *Monilinia* species. Furthermore, the biocontrol strain of *Trichoderma* T6 presented significant antagonistic activity on the *M. yunnanensis* T8-1 isolate (T8-1) among the five *Trichoderma* strains. The highest inhibitory rates for *Trichoderma* T6 and its fermentation product against T8-1 mycelial growth were 72.13% and 68.25%, respectively. The obvious inhibition zone displayed on the colony interaction area between the colony of T8-1 isolate and *Trichoderma* T6 and the morphological characterization of the T8-1 hyphae were enlarged and malformed after inoculation with the *Trichoderma* T6 fermentation product at 20-fold dilution. Our results indicate that the strain of *Trichoderma* T6 could be considered as a beneficial biocontrol agent in managing brown rot of peach fruit disease.

## 1. Introduction

Peach (*Prunus persica* (L.) Batsch) is one of the most important global tree crops within the economically important Rosaceae family, and an important deciduous fruit tree used for worldwide cultivation in temperate and subtropical zones [1]. According to the Food and Agriculture Organization’s statistics, peach trees produce more than 24 million metric tons of fruit per year [2]. China is the largest peach-producing country in the world, with 15.02 million tons of fruit annually in 2020, followed by Spain (1.31 million tons), Italy (1.02 million tons), Turkey and Greece (0.89 million tons each), Iran (0.66 million tons), and the USA (0.56 million tons) [3]. However, peach production is negatively influenced by pre-harvest and post-harvest diseases during the growth, storage, and subsequent shelf-life stages [4]. The brown rot caused by *Monilinia* spp. represents one of the most economically important and destructive diseases in peach fruit worldwide [5].

Peach brown rot usually appears as soft brown spots on the fruit in the initial stage, and then produced a greyish powdery mass of conidia on the surfaces of the fruit samples after being infected by *Monilinia* spp. pre-harvest and post-harvest. The species and distribution of the *Monilinia* pathogens that cause brown rot on peaches have been reported previously. *Monilinia fructicola* (G. Winter), *M. laxa* (Aderhold and Ruhland), and *M. fructigena* (Honey) are three main species found in peach orchards worldwide [6,7]. *Monilinia fructicola* is one of the most important causal agents of brown rot of peach fruit [8] and is found in peach orchards in Turkey [9], Brazil [10], South Carolina [11], Georgia [12], Spain [13], Italy [14], Czech Republic [15], Slovenia [16], China [6,17,18], Canada [19], Australia [20], and New Zealand [21], causing significant yield losses and damages in the field and during storage and distribution to market. Obi et al. (2018) reported that more than 50% of the global post-harvest peach losses are due to the brown rot disease [5]. *Monilinia laxa* is another causal agent of brown rot in peach orchards in Turkey [9], Italy [22], and Spain [23,24], whereas *M. fructigena* is found and detected in peach orchards in Italy [22]. In China, *M. fructicola*, *Monilia mumecola,* and *M. yunnanensis* are the main agents of brown rot [25]. In 2017–2018, approximately 40% of peaches were affected annually by the brown rot disease in peach orchards in Nyingchi and Qamdo, Tibet. The brown rot disease has now become one of the main factors that restricts the yield and quality of peach fruit production and has caused severe economic losses in Tibet due to the changed climate and ineffective management strategies. Therefore, it is urgent to identify the pathogen that causes brown rot in peaches in Tibet and to find effective biocontrol agents to control peach fruit diseases.

Sequence and phylogenetic analyses of the internal transcribed spacer (ITS) region have been widely applied and performed in terms of the taxonomy and molecular phylogeny [26]. For example, they were used for the identification of *Monilinia* species on the fruit of *Crataegus* spp. and *Rhododendron* spp. hosts [27,28]. The phylogenies of *TUB2* and *G3PDH* sequences have also revealed significant taxonomic relations in fungi [29,30,31]. In addition, a new multiplex PCR method was developed to facilitate the detection of *M. yunnanensis* and the differentiation of *Monilinia* spp. causing brown rot in peaches in China [25]. However, little is known about the specific species that cause the brown rot disease of peaches in Nyingchi and Qamdo, Tibet.

*Trichoderma* spp. are well-known beneficial isolates for the antagonistic abilities against many plant pathogens due to their mycoparasitic, reproductive, and competitive capacity [32,33]. Some previous studies have demonstrated that the species of *Trichoderma* exhibited a wide range of antagonistic abilities against plant pathogens, including *Fusarium oxysporum* [34,35,36], *Alternaria porri* [37], *Verticillium dahliae* [38], *Rhizoctonia solani* [39], *Botrytis cinerea* [40,41], *Fusarium solani* RC 386 [42], *Colletotrichum capsici*, and *M. fructicola* [43]. In addition, the fungi of *Epicoccum nigrum* [44], *Penicillium frequentans* [45], *Candida pruni* sp. nov. [46], and *Trichothecium roseum* [47] have been identified as biocontrol agents in controlling the brown rot disease of stone fruits. However, there is little information regarding the efficacy of *Trichoderma* spp. against the pathogen of peach brown rot disease in Tibet. The aims of our present study are to (i) survey and describe the *Monilinia* species infecting the peaches in the primary fruit production regions of Tibet; (ii) carry out a morphological investigation and molecular analysis for the identification of *Monilinia* species; and (iii) determine the biocontrol activity of *Trichoderma* strains and their fermentation products against the pathogens isolate in vitro. Our findings would provide a solid basis for making scientific and effective management strategies for the brown rot disease of peach fruit.

## 2. Material and Methods

### 2.1. Sample Collection and Pathogen Isolation

Peach (*Prunus persica* (L.) Batsch) fruit samples were collected from 11 peach orchards in the Nyingchi and Qamdo regions, Tibet. Five trees per peach orchard were investigated, and a total of 23 peach fruit samples with symptoms of brown lesions covered by grayish brown sporodochia were used to isolate the pathogens. Nyingchi is in the southeast of Tibet, at the longitude of 94.362348 E and latitude of 29.654693 N. The average altitude, temperature, and precipitation are about 3000 m, 8–10 °C, and 600–1000 mm, respectively. Qamdo is seated in the eastern part of the Tibet autonomous region, southwest China, at the longitude of 97.1699 E and latitude of 31.145 N. The average altitude, temperature, and precipitation were about 3500 m, 7–8 °C, and 400–600 mm, respectively. The amount of rotten fruit with characteristic sporulation of the pathogen was recorded at harvest by determining the incidence of peach brown rot [48]. The average disease incidence was more than 40% in 2017–2018.

For the identification of *Monilinia* spp., five small fruit sections (0.5 cm × 0.5 cm) with brown rot symptoms from 23 fruit samples were superficially disinfected with 75% ethanol for 1 min and then with 1% sodium hypochlorite (NaClO) for 1 min, followed by rinsing in sterile distilled water three times. The sterilized fruit sections were dried on sterile paper and placed individually in Petri dishes containing potato dextrose agar (PDA), then the dishes were incubated at 22 °C for 5 days.

### 2.2. Purification of Isolates

For each isolate, a piece of agar (5 mm in diameter) with mycelia was cut from the edge of a 5-day-old colony and placed upside down onto the center of a fresh PDA medium, then the medium incubated at 22 °C in darkness. Twenty-three isolates were examined morphologically according to Lane (2002) [49]. The isolates with different morphological characteristics were inoculated into peach fruit samples and used as the representative subsamples of single spore isolates for further morphological and molecular characterizations to identify the species. The single-spore isolation process was conducted as described previously by Luo et al. (2002) [50].

### 2.3. Morphological Observations of Colonies and Conidial Spores

The morphological characteristics (shape, color, stromata) of re-isolated single spores of 23 isolates were observed after inoculation on PDA media. The colony diameter was measured in two perpendicular directions with six replicates. The growth of the obtained colonies on PDA media was recorded, and the average daily growth rate was calculated. The conidia were collected from the lesions developed on peach fruit samples with a gray powdery mass of conidia. The morphological characteristics of the conidia and the conidial germination pattern were observed under a light microscope (E200, Beijing, China). Additionally, the sizes (width and length) of 50 conidia samples were measured for each of the 6 replicates, and the mean value was determined for each isolate.

### 2.4. Pathogenicity Testing of Isolates

The pathogenicity was tested on the detached and healthy peach fruit samples by inoculating a mycelial plug of the isolates on the surfaces of disinfected mature peach fruit samples. Briefly, healthy peach fruit samples were superficially disinfected with 75% ethanol for 1 min, then rinsed with sterile water three times and air-dried at 22 ± 2 °C prior to inoculation. A 5-mm-diameter plug with active mycelium from a 5-day-old culture of the isolate was inoculated into a 5-mm-diameter wound of a healthy peach. Thereafter, the inoculated fruit samples were placed on a metal support in a plastic container (28.50 cm × 18.5 cm × 9 cm) with sterile water at the bottom. The plastic container was then covered with cling film and incubated at 22 °C with a 12 h/12 h light/dark cycle. The pathogenicity was determined from 2 to 6 days after the inoculation for 1 day internally. At day 6, the infected fruit sample was re-isolated and used to identify the isolates using morphological and molecular characteristics [25]. The healthy peach fruit inoculated with a sterile plug without a mycelium served as the control. All experiments were repeated twice, and three inoculated and control fruit samples were used in one replicate for the treatment and control, respectively.

### 2.5. Molecular Identification of Representative Isolates

To confirm the morphological identification, genomic DNA samples of the mycelia of pure isolates with the typical brown rot symptoms were extracted using the modified CTAB method. The internal transcribed spacer (ITS) regions 1 and 2, glyceraldehyde-3-phosphate dehydrogenase (*G3PDH*), and β-tubulin (*TUB2*) gene fragments were amplified via PCR using the primers ITS1/ITS14, Mon-G3pdhF/Mon-G3pdhR, and Mon-TubF1/Mon-TubR1 of Hu et al. (2011) [25]. The PCR reaction was carried out with a T100™ Thermal Cycler (Bio-Rad, California, USA). The PCR products of the ITS region, *G3PDH,* and *TUB2* genes fragments were sent to Sangon Biotech (Shanghai, Co., Ltd., China) for sequencing. The sequences of the *G3PDH* and *TUB2* genes were aligned using DNAman version 8.0 and used to construct the phylogenetic trees with the neighbor-joining (NJ) method using MEGA version 10.0 with default parameters and 1000 bootstrapping replicates. In addition, a multiplex PCR protocol and reaction were used to identify the representative isolates, which was developed previously to identify the three *Monilia* species on peaches in China [25].

### 2.6. Biocontrol Activity of Trichoderma Strains against the Pathogen of T8-1

#### 2.6.1. *Trichoderma* Strain Preparation and Antagonistic Activity Determination

Five strains of *Trichoderma* (T6, B3, D5, D6, and J1) were isolated from the soil in Gansu, China, and used in the present study to determine its antifungal potential against the pathogen of the T8-1 isolate in in vitro experiments. The active colony was then prepared via culturing on PDA in Petri dishes for 6 days at 25 °C. The antagonistic activity of the *Trichoderma* strains against the pathogen T8-1 was assessed following the dual culture plate technique. Each experiment involved six replications. The dishes were incubated at 25 °C for 6 days with supplemental day/night lighting of 16/8 h. The inhibitory rate was calculated according to the formula described by Etebarian et al. (2005) [51] at 6 days after inoculation.

Inhibitory rate (%) = ((Colony radius in control group − Colony radius in treatment group)/(Colony radius in control group − 0.25)) ×100%

#### 2.6.2. *Trichoderma* Strain Fermentation and Antagonistic Activity Determination

The *Trichoderma* strains were fermented according to the method described by Zhang et al. (2018) [52]. The fermentation filtrates were then diluted to 20-, 40-, 80-, 160-, and 320-fold with sterile water. For the determination of the antagonistic activity levels of the different dilutions of *Trichoderma* strain fermentations against the pathogen of the T8-1 isolate, a 0.25-cm-radius plug was collected from a 5-day-old mycelial culture of the T8-1 isolate and placed in the center of fresh PDA medium with 2 mL of *Trichoderma* T6 fermentation (20- to 320-folds). The PDA medium inoculated with equal volumes of sterile water and T8-1 isolate (without *Trichoderma* T6 fermentation) was used as the control. The dishes were incubated at 25 °C for 7 days with supplemental day/night lighting of 16/8 h. The inhibitory rate was calculated according to the formula described by Etebarian et al. (2005) [51] at 7 days after inoculation. The morphological characterization of T8-1 hyphae was observed using the microscope observation after inoculation with *Trichoderma* T6 fermentation (20-fold) at 7 days. The entire experiment had six replications. 

### 2.7. Statistical Analysis

The data analyses were performed with SPSS statistics 20.0 and analyzed via a one-way ANOVA. A comparison between the means was performed using Duncan’s multiple range test and the T values of the adjusted degrees of freedom. The differences were considered significant at *p < 0.05*.

## 3. Results

### 3.1. Symptoms of Peach Brown Rot in Fields

**Figure 1:**
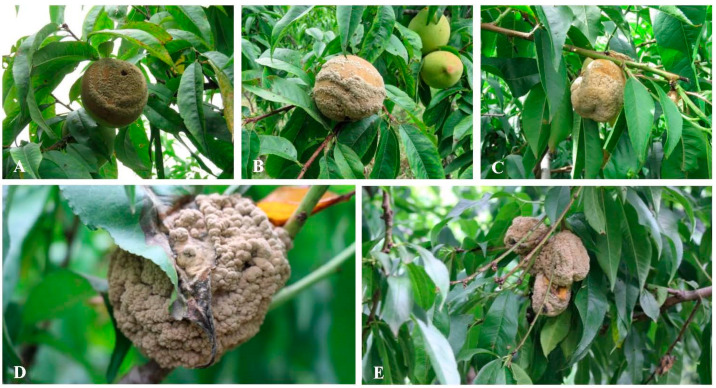
Symptoms of peach brown rot caused by *Monilia yunnanensis* in the field in Tibet, China: (**A**–**C**) during the growing season; (**D**,**E**) at harvest time.

Our field investigation found that the fruit infection appeared as soft brown spots that rapidly produced a greyish powdery mass of conidia, often in concentric rings on the rotted areas during the growing period (Figure 1A–C). The infected fruit eventually dried, shriveled into wrinkled mummies and attached to the tree until harvest (Figure 1D,E).

### 3.2. Isolates Purification

At day 5, 23 pure-culture isolates with the same characteristics were isolated and coded as T8-1 to T8-23 for hyphal tip isolation and further experimentation. The colony colors of the isolates were white to gray-green with even concentric rings and margins. The average daily growth rate of the obtained colonies on the PDA media was 10.52 mm. T8-1, T8-8, and T8-20 were isolated from different years and regions and used to ascertain the pathogen species based on morphological and molecular characteristics.

### 3.3. Characteristics of Single Spores of the Representative Isolate T8-1

**Figure 2:**
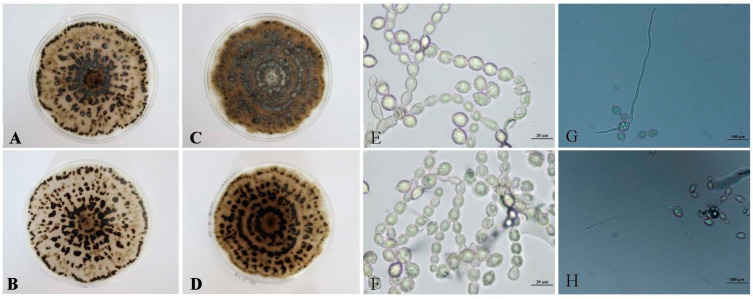
Characteristics of a single-spore isolate of *Monilia yunnanensis* (isolate T8-1) grown on PDA medium at 22 °C for 13 and 16 days: (**A**,**B**) front and reverse views of T8-1 colonies at 13 days after inoculation, respectively; (**C**,**D**) front and reverse colonies of T8-1 colonies at 16 days after inoculation, respectively; (**E**,**F**) the conidial morphology characteristics of T8-1; (**G**,**H**) the conidial germ tube morphology characteristics of T8-1.

After 13 days of incubation at 22 °C on PDA medium, the T8-1, T8-8, and T8-20 pure-culture isolate colonies had same characteristics. The color of the representative isolate T8-1 colony changed from white to grayish, then the stromata developed (Figure 2A,B) and the color of the colony changed from grayish to brown to black. Abundant black stromata developed after 16 days of incubation (Figure 2C,D). The conidia were hyaline, one-celled, mostly lemon-shaped, and formed branched chains on the inoculated peach fruit samples. The average diameters of the conidia were 11.11 to 19.21 μm (mean = 15.16 μm) × 8.18 to 13.52 μm (mean = 10.85 μm) (Figure 2E,F). Additionally, we found that two germ tubes were often produced from the pointy sides of the conidia (Figure 2G,H). 

### 3.4. Pathogenicity Test on Peach Fruit Samples at Different Days after Inoculation 

**Figure 3:**
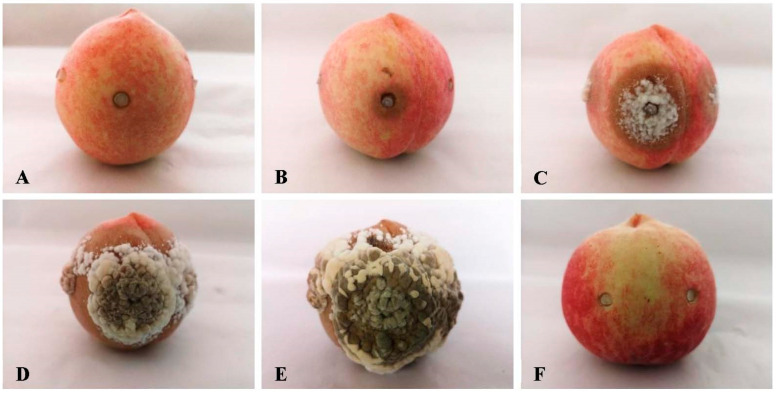
Symptoms from the pathogenicity tests on peach fruit samples at different days after inoculation with the isolate T8-1: (**A**–**E**) the symptoms at 2, 3, 4, 5, and 6 days after inoculation with the T8-1 isolate; (**F**) the control peach at 6 days after inoculation with sterile PDA plugs without the T8-1 isolate (control).

After 2–3 days of incubation of the inoculated fruit samples with T8-1, T8-8, and T8-20 at 22 °C, the T8-1 isolate had high pathogenicity. The typical brown rot symptoms appeared (Figure 3A) and the size of the rot area increased as the incubation time increased with the representative isolate T8-1 (Figure 3B). Thereafter, the fruit infections appeared as soft brown spots that rapidly produced white hyphae at day 4 (Figure 3C), and then at day 5 a greyish powdery mass of conidia formed in the concentric rings on the rotted areas (Figure 3D). At day 6, all inoculated fruit samples developed brown rot symptoms that were similar to those observed in the fields (Figure 3E), while no symptoms were observed on the control fruit samples (Figure 3F).

### 3.5. Molecular Identification of Representative Isolates

**Figure 4:**
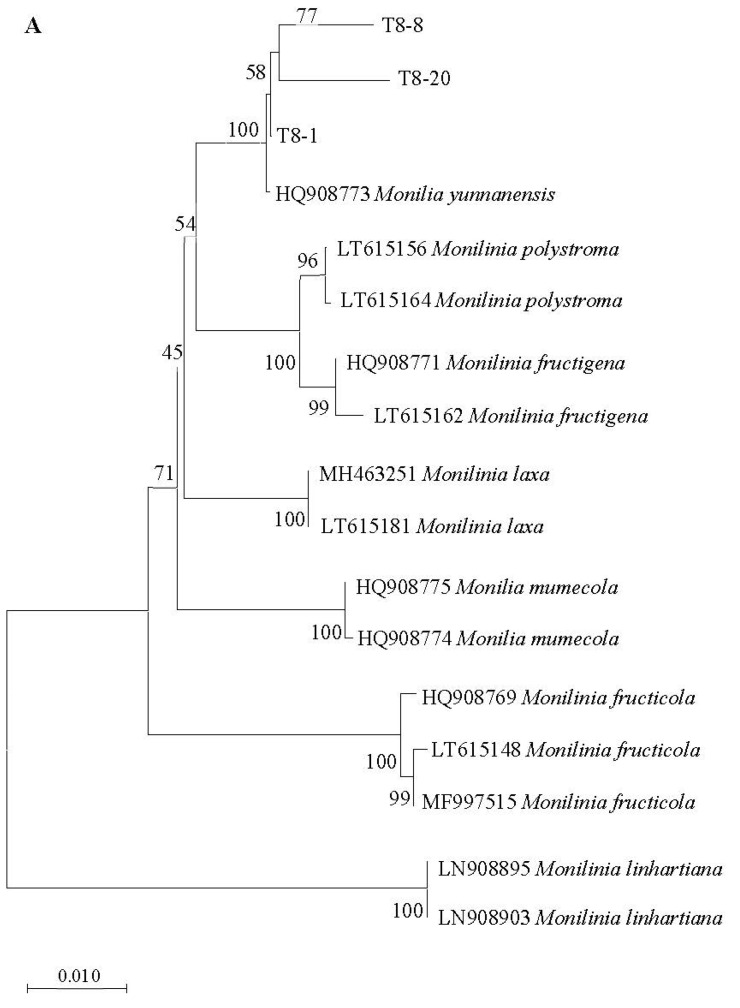

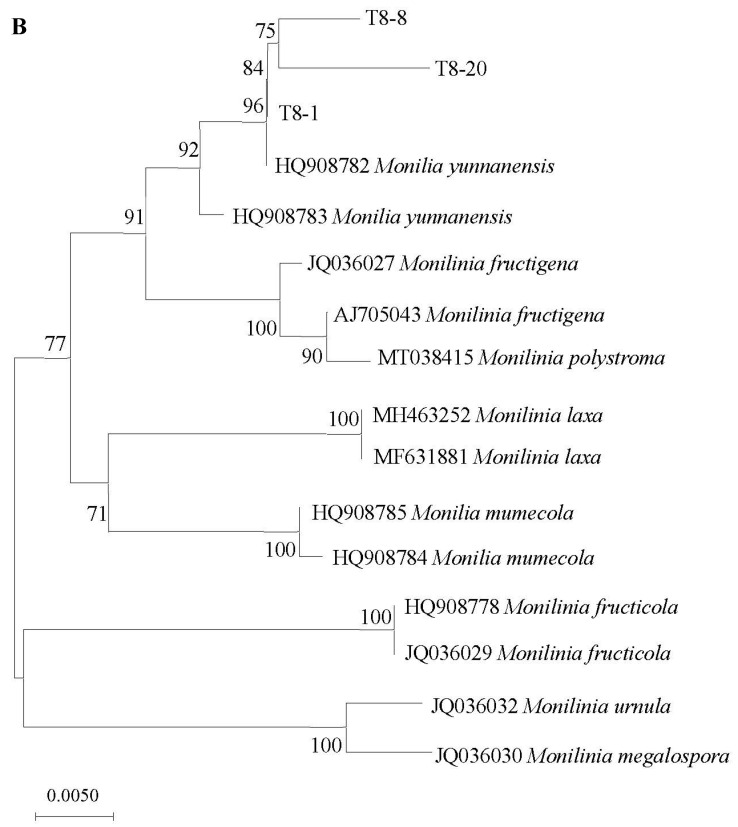
Phylogenetic trees of the representative isolates T8-1, T8-8, and T8-20 were inferred from sequences of *TUB2* (**A**) and *G3PDH* (**B**). The photogenic trees were formed with the neighbor-joining (NJ) method using MEGA version 10.

**Figure 5:**
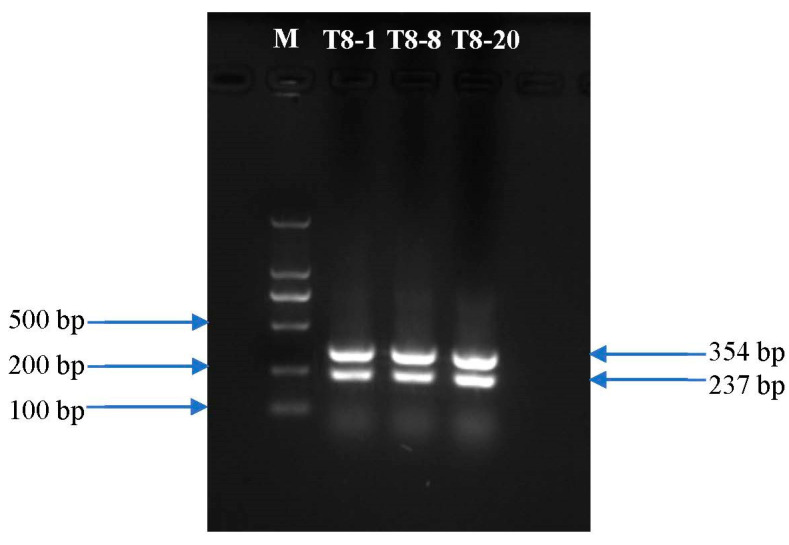
Multiplex polymerase chain reaction (PCR) results for the representative isolates T8-1, T8-8, and T8-20. Lane M, marker.

The consensus sequence of the ITS regions of the T8-1, T8-8, and T8-20 isolates were 99.40 to 99.79% and 98.58 to 98.79% identical to those of *M. fructicola* (accession no. KF516935) and *M. yunnanensis* (accession no. MW355895), respectively. The sequences of the T8-1, T8-8, and T8-20 *TUB2* and *G3PDH* genes had higher identity rates of 99.00 to 99.93% and 98.48 to 99.86% to the *TUB2* and *G3PDH* sequences of *M. yunnanensis* (accession no. HQ908773 and HQ908782), respectively, than those of other *Monilinia* species, and formed a monophyletic clade with those of *M. yunnanensis* (HQ908783 and HQ908782) in the *TUB2* and *G3PDH* phylogenetic trees, with bootstrapping support values of 0.01 and 0.005, respectively (Figure 4A,B). The results of the multiplex PCR detection test showed that a 354 bp fragment was amplified from the T8-1, T8-8, and T8-20 isolates, indicating that the T8-1, T8-8, and T8-20 isolates were from the *Monilia* genus. Additionally, a fragment of 237 bp was amplified from the T8-1, T8-8, and T8-20 isolates, which indicated them as *M. yunnanenis* (Figure 5). From the results of the multiplex PCR detection and ITS region tests, and the *TUB2* and *G3PDH* genes sequencing confirmed that the T8-1, T8-8, and T8-20 isolates were from *M. yunnanensi.*

### 3.6. Biocontrol Activity of Trichoderma Strains against the Pathogen of the M. yunnanenis T8-1 Isolate

**Figure 6:**
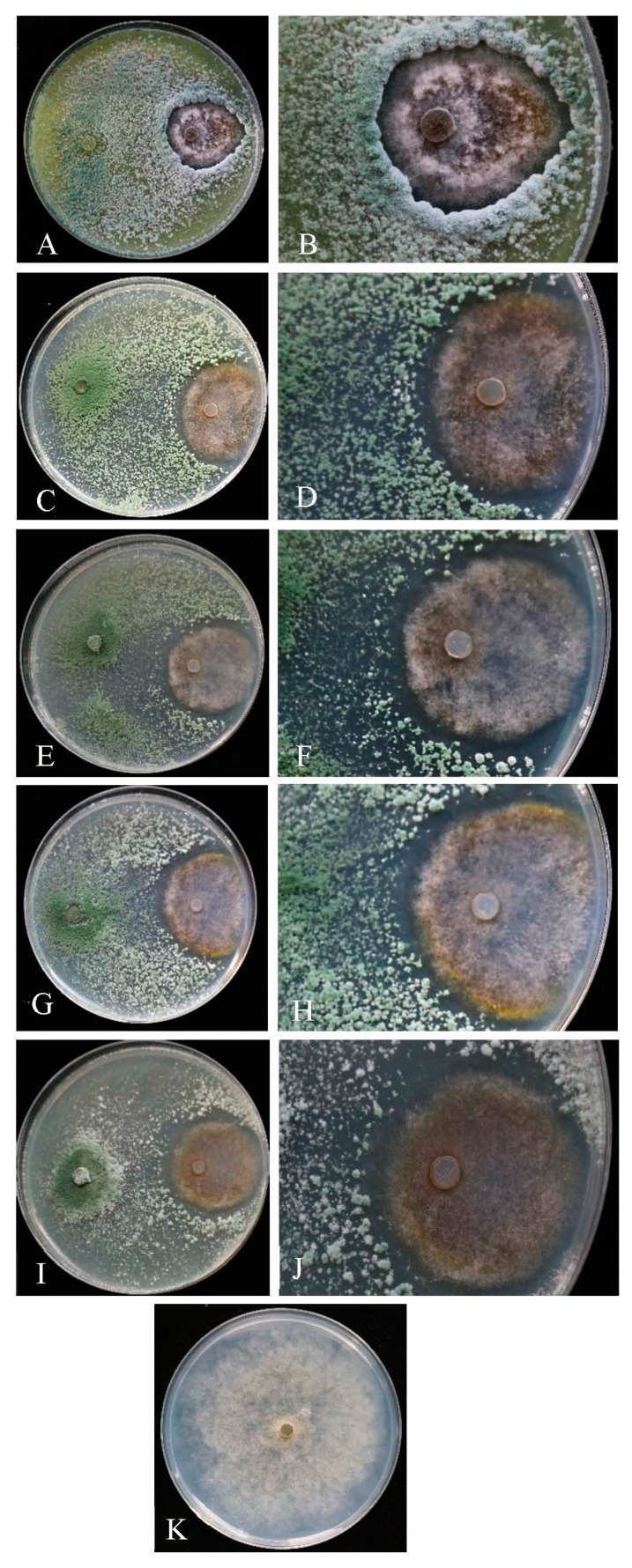
Antagonistic activity levels of five *Trichoderma* strains against *Monilia yunnanensis* after dual culture at 6 days: (**A**,**C**,**E**,**G**,**I**) the antagonistic activity levels of *Trichoderma* strains T6, B3, D5, J1, and D6 against *M. yunnanensis* in dual culture, respectively; (**B**,**D**,**F**,**H**,**J)** the interactions of *Trichoderma* T6, B3, D5, J1, and D6 colonies with *M. yunnanensis*, respectively; (**K)** the normal colony of *M. yunnanensis* (control).

Compared to the control (Figure 6K), different strains of *Trichoderma* exhibited the significant antagonistic activity on the pathogen of *M. yunnanenis* T8-1 isolate (T8-1). The colony radius of the T8-1 isolate was significantly inhibited at 6 days after inoculation with the *Trichoderma* strains T6 (Figure 6A), B3 (Figure 6C), D5 (Figure 6E), J1 (Figure 6G), and D6 (Figure 6I). The colony interaction area showed an obvious inhibition zone between the colonies of the *Trichoderma* strains T6 (Figure 6B), B3 (Figure 6D), D5 (Figure 6F), J1 (Figure 6H), and D6 (Figure 6J) and the T8-1 isolate, respectively. The T8-1 isolate colony color changed to brown in comparison to the control. The inhibitory rates of the T8-1 isolate were 72.13%, 63.67%, 62.57%, 64.48%, and 61.75% at 6 days after inoculation with the *Trichoderma* strains T6, B3, D5, J1, and D6, respectively (Table 1). The *Trichoderma* strain T6 had a significant inhibitory effect on the T8-1 isolate in comparison to the other four strains (Table 1).

### 3.7. Biocontrol Activity and Morphological Characterization of Trichoderma T6 Fermentation against the Pathogen of the M. yunnanenis T8-1 Isolate

**Figure 7:**
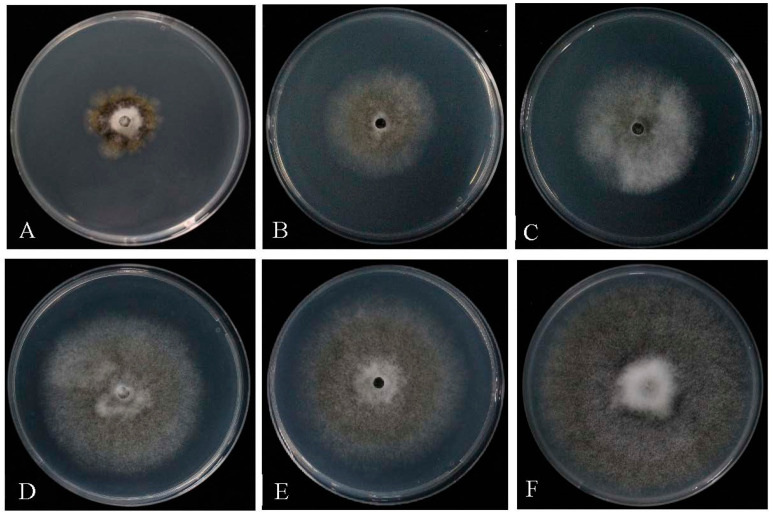
Antagonistic activity levels of *Trichoderma* T6 fermentation against *Monilia yunnanensis* after inoculation at 7 days: (**A**–**F**) the antagonistic activity levels of 20-, 40-, 80-, 160-, and 320-fold fermentation dilutions and the sterile water (control), respectively.

**Figure 8:**
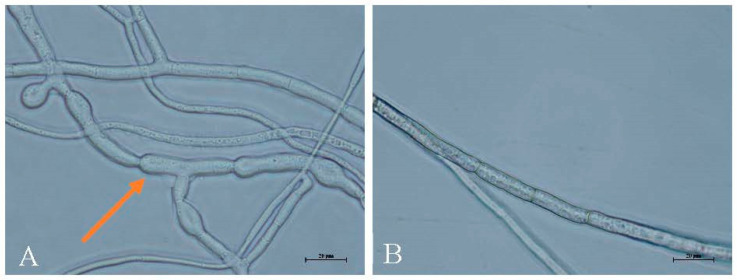
Morphological characterization of *Monilia yunnanensis* hyphae after inoculation with *Trichoderma* T6 fermentation (20-fold) at 7 days: (**A**) the hyphae of *M. yunnanensis* was enlarged and malformed after inoculation with *Trichoderma* T6 fermentation; (**B**) the normal hyphae of *M. yunnanensis* in the control group after inoculation with sterile water (Control). The arrow represents the enlarged and malformed hyphae.

Different dilutions of the *Trichoderma* T6 fermentation exhibited significant antagonistic activity on the pathogen of the *M. yunnanenis* T8-1 isolate. The inhibitory rates of the T8-1 isolate were increased with decreasing *Trichoderma* T6 fermentation dilutions. The colony radius of the T8-1 isolate was significantly inhibited at 7 days after being inoculated with 20- (Figure 7A), 40- (Figure 7B), 80- (Figure 7C), 160- (Figure 7D), and 320-fold (Figure 7E) *Trichoderma* T6 fermentation dilutions in comparison to the control (Figure 7F). The inhibitory rates of the T8-1 isolate were 68.25%, 50.00%, 41.00%, 27.00%, and 18.00% at 7 days after inoculation with 20-, 40-, 80-, 160-, and 320-fold *Trichoderma* T6 fermentation dilutions, respectively (Table 2). In addition, the morphological characterization of the T8-1 isolate hyphae was enlarged and malformed after inoculation with the *Trichoderma* T6 fermentation product (20-fold dilution) (Figure 8A) at 7 days, whereas the normal hyphae of the T8-1 isolate were observed in the control group (Figure 8B).

## 4. Discussion

Our present study reports the first record of a *Monilia* species causing brown rot in peaches in the Nyingchi and Qamdo regions, Tibet. Based on the morphological characterization and molecular identification, the representative isolates of T8-1, T8-8, and T8-20 were identified as *M. yunnanensis*. Meanwhile, we reported that *M. yunnanensis* can cause brown rot in nectarines in Nyingchi, Tibet [53]. However, there is no information regarding the *Monilia* species causing brown rot in peaches in the Nyingchi and Qamdo regions. To our knowledge, this is the first report of *M. yunnanensis* causing brown rot in peaches in Nyingchi and Qamdo, Tibet.

The morphological characteristics in terms of the spore size, colony morphology, growth rate, and pathogenicity of the isolates T8-1, T8-8, and T8-20 on the peach fruit samples are very similar to those of *M. yunnanensis. Monilia yunnanensis* was first reported as a new fungal species causing peach brown rot in Yunnan province, China [25]. Since then, it has been reported as the pathogenic agent of brown rot on hawthorn [54], plum [55], and apricot [56] samples in China.

The biological-morphology-based species identification results were subsequently confirmed by the multiplex PCR method, showing the T8-1, T8-8, and T8-20 isolates as *M. yunnanenis*. Furthermore, the phylogenetic analysis of the ITS sequences indicated that the isolates T8-1, T8-8, and T8-20 are most closely related to *M. fructicola* and *M. yunnanensis*, whereas the *G3PDH* and *TUB2* sequences are most closely related to *M. yunnanensis*. However, the morphological characteristics are significantly different between *M. fructicola* and *M. yunnanensis.* The colonies of *M. fructicola* from peach and plum samples are brown with fluffy mycelia and abundant mycelial tufts of sporulation [6,54]. In contrast, the colony of *M. yunnanensis* is gray-greenish and covered with whitish felty mycelia and minor exceptions of lobbed margins. *Monilia yunnanensis* also has no mycelial tufts of sporulation [25,55,56]. Therefore, the representative isolates of T8-1, T8-8, and T8-20 were identified as *M. yunnanensis* based on both their morphological and molecular characteristics.

Furthermore, our results showed that the strain of *Trichoderma* T6 and its fermentation presented significant antagonistic activity on the pathogen of the *M. yunnanenis* T8-1 isolate (T8-1). A previous study reported that *T. harzianum* ITEM 3636 can inhibit the pathogen of peanut brown root rot (*F. solani* RC 386) in terms of mycelial growth. The highest inhibition growth percentages for *T. harzianum* ITEM 3636 and its filtered liquid cultures were 48.4% and 78.2%, respectively [42]. Similarly, the inhibition effects of *T. afroharzianum* TM24 on *B. cinerea*, *F. oxysporum* f. sp. *cucumerinum*, *C. capsici*, *M. fructicola,* and *F*. *oxysporum* f. sp. *niveum* reached 74.2%, 43.4%, 51.9%, 66.7%, and 51.0%, respectively [43]. Our results revealed that the highest inhibitory rates of *Trichoderma* T6 and its fermentation product against T8-1 isolate mycelial growth were 72.13% and 68.25%, respectively. Fu et al. (2017) reported that the antagonistic activity of the natural compound berberine on *M. fructicola* was 72.7% at 23.44 µg/mL [57]. The result from our present study shows no significant difference in comparison to the biocontrol agent of berberine. In addition, we found an obvious inhibition zone displayed on the colony interaction area between the colony of the T8-1 isolate and *Trichoderma* T6, and even the enlarged and malformed hyphae of the T8-1 isolate was observed after inoculation with *Trichoderma* T6 fermentation.

## 5. Conclusions

In the present study, we identified *M. yunnanensis* in peach orchards in the regions of Nyingchi and Qamdo, Tibet. The strain *Trichoderma* T6 could be considered as a beneficial biocontrol agent in controlling peach brown rot disease. The future research will focus on the biocontrol mechanisms of *Trichoderma* T6 and its fermentation product against peach brown root rot caused by *M. yunnanensis*.

## Figures and Tables

**Table 1:** Inhibitory activity of *Trichoderma* strains on the growth of the peach brown rot pathogen of the *Monilia yunnanensis* T8-1 isolate.

Trichoderma Strains	Colony Radius (cm)		Inhibitory Rates (%)
	Treatment	Control	
T6	1.27	3.91	72.13 ± 1.41 a
B3	1.58	3.91	63.67 ± 0.73 b
D5	1.62	3.91	62.57 ± 0.79 b
J1	1.55	3.91	64.48 ± 0.72 b
D6	1.65	3.91	61.75 ± 1.43 b

The data are means ± standard errors of replicates, and those in a column followed by different letters are significantly different at *p < 0.05*, based on Duncan’s new multiple range test using a one-way ANOVA (n = 6). The inhibitory rates (%) were determined at 6 days after inoculation with *M. yunnanensis*. The control represents the media inoculation with sterile water.

**Table 2:** Inhibitory activity levels of *Trichoderma* T6 fermentation products on the growth of the *Monilia yunnanensis* T8-1 isolate.

Dilution Folds (Folds)	Colony Radius (cm)		Inhibitory Rates (%)
	Treatment	Control	
20	1.52	4.25	68.25 ± 0.95 a
40	2.25	4.25	50.00 ± 1.03 b
80	2.61	4.25	41.00 ± 1.85 c
160	3.17	4.25	27.00 ± 1.35 d
320	3.53	4.25	18.00 ± 1.65 e

The data are means ± standard errors of replicates, and those in a column followed by different letters are significantly different at *p* < 0.05, based on Duncan’s new multiple range test using a one-way ANOVA. The inhibitory rates (%) were determined at 7 days after inoculation with *M. yunnanensis*. The control represents the media inoculation with sterile water.

## Data Availability

The data in this study are available on request from the corresponding author or first author.

## References

[1] FAOSTAT (2020). Food and Agricultural Organization of the United Nations. https://www.statista.com/statistics/264001/worldwide-production-of-fruit-by-variety/.

[2] FAOSTAT (2017). Food and Agricultural Organization of the United Nations. http://www.fao.org/faostat/en/#data.

[3] FAOSTAT (2020). Food and Agricultural Organization of the United Nations. https://www.fao.org/faostat/en/#data/QCL.

[4] Sisquella M., Viñas I., Picouet P., Torres R., Usall J. (2014). Effect of host and *Monilinia* spp. variables on the efficacy of radio frequency treatment on peaches. Postharvest Biol. Tec..

[5] Obi V.I., Barriuso J.J., Gogorcena Y. (2018). Peach brown rot: Still in search for an ideal management option. Agriculture.

[6] Hu M.J., Chen Y., Chen S.N., Liu X.L., Yin L.F., Luo C.X. (2011). First report of brown rot of peach caused by *Monilinia fructicola* in southeastern China. Plant Dis..

[7] Zhu X.Q., Zheng H.H., Fang Y.L., Guo L.Y. (2014). A method to induce significant production of conidia from *Monilinia fructigena*, *Monilia polystroma*, and *Monilia yunnanensis*. Australas. Plant Pathol..

[8] Zhu X.Q., Chen X.Y., Luo Y., Guo L.Y. (2005). First report of *Monilinia fructicola* on peach and nectarine in China. Plant Pathol..

[9] Ozkilinc H., Yildiz G., Silan E., Arslan K., Guven H., Altinok H.H., Altindag R., Durak M.R. (2020). Species diversity, mating type assays and aggressiveness patterns of *Monilinia* pathogens causing brown rot of peach fruit in Turkey. Eur. J. Plant Pathol..

[10] Fischer J.M.M., Savi D.C., Aluizio R., De Mio M.L.L., Glienke C. (2017). Characterization of *Monilinia* species associated with brown rot in stone fruit in Brazil. Plant Path..

[11] Landgraf F.A., Zehr E.I. (1982). Inoculum sources for *Monilinia fructicola* in South Carolina peach orchards. Phytopathology.

[12] Emery K.M., Michailides T.J., Scherm H. (2000). Incidence of latent infection of immature peach fruit by *Monilinia fructicola* and relationship to brown rot in Georgia. Plant Dis..

[13] de Cal A., Gell I., Usall J., Viñas I., Melgarejo P. (2009). First report of brown rot caused by *Monilinia fructicola* in peach orchards in Ebro Valley, Spain. Plant Dis..

[14] Pellegrino C., Gullino M.L., Garibaldi A., Spadaro D. (2009). First report of brown rot of stone fruit caused by *Monilinia fructicola* in Italy. Plant Dis..

[15] Duchoslavová J., Širučková I., Zapletalová E., Navrátil M., Šafářová D. (2007). First report of brown rot caused by *Monilinia fructicola* on various stone and pome fruits in the Czech Republic. Plant Dis..

[16] Munda A., Viršček M.M. (2010). First report of brown rot caused by *Monilinia fructicola* affecting peach orchards in Slovenia. Plant Dis..

[17] Zhong Y.F., Zhang Y.W., Chen X.Y., Luo Y., Guo L.Y. (2008). Overwintering of *Monilinia fructicola* in stone fruit orchards in northern China. J. Phytopathol..

[18] Yin L.F., Chen G.K., Chen S.N., Du S.F., Li G.Q., Luo C.X. (2014). First report of brown rot caused by *Monilia mumecola* on Chinese sour cherry in Chongqing Municipality, China. Plant Dis..

[19] Biggs A.R., Northover J. (1988). Early and late-season susceptibility of peach fruits to *Monilinia fructicola*. Plant Dis..

[20] Tran T.T., Li H., Nguyen D.Q., Sivasithamparam K., Jones M.G.K., Wylie S.J. (2017). Spatial distribution of *Monilinia fructicola* and *M. laxa* in stone fruit production areas in Western. Australas. Plant Pathol..

[21] Elmer P.A.G., Spiers T.M., Wood P.N. (2007). Effects of pre-harvest foliar calcium sprays on fruit calcium levels and brown rot of peaches. Crop Prot..

[22] Abate D., Pastore C., Gerin D., De Miccolis Angelini R.M., Rotolo C., Pollastro S., Faretra F. (2018). Characterization of *Monilinia* spp. populations on stone fruit in South Italy. Plant Dis..

[23] Gell I., Larena I., Melgarejo P. (2007). Genetic diversity in *Monilinia laxa* populations in peach orchards in Spain. J. Phytopathol..

[24] Gell I., De Cal A., Torres R., Usall J., Melgarejo P. (2008). Relationship between the incidence of latent infections caused by *Monilinia* spp. and the incidence of brown rot of peach fruit: Factors affecting latent infection. Eur. J. Plant Pathol..

[25] Hu M.J., Cox K.D., Schnabel G., Luo C.X. (2011). *Monilinia* species causing brown rot of peach in China. PLoS ONE.

[26] Iwen P.C., Hinrichs S.H., Rupp M.E. (2002). Utilization of the internal transcribed spacer regions as molecular targets to detect and identify human fungal pathogens. Med. Mycol..

[27] Holst-Jensen A., Kohn L., Jakobsen K., Schumacher T. (1997). Molecular phylogeny and evolution of *Monilinia* (Sclerotiniaceae) based on coding and noncoding rDNA sequences. Am. J. Bot..

[28] Takahashi Y., Ichihashi Y., Sano T., Harada Y. (2005). *Monilinia jezoensis* sp. nov. in the Sclerotiniaceae, causing leaf blight and mummy fruit disease of *Rhododendron kaempferi* in Hokkaido, northern Japan. Mycoscience.

[29] Fournier E., Giraud T., Albertini C., Brygoo Y. (2005). Partition of the *Botrytis cinerea* complex in France using multiple gene genealogies. Mycologia.

[30] O’Gorman D.T., Sholberg P.L., Stokes S.C., Ginns J. (2008). DNA sequence analysis of herbarium specimens facilitates the revival of *Botrytis mali*, a postharvest pathogen of apple. Mycologia.

[31] Staats M., van Baarlen P., van Kan J.A.L. (2005). Molecular phylogeny of the plant pathogenic genus *Botrytis* and the evolution of host specificity. Mol. Biol. Evol..

[32] Hermosa R., Viterbo A., Chet I., Monte E. (2012). Plant-beneficial effects of *Trichoderma* and of its genes. Microbiology.

[33] Lopes F.A.C., Steindorff A.S., Geraldine A.M., Brandao R.S., Monteiro V.N., Júnior M.L., Coelho A.S.G., Ulhoa C.J., Silva R.N. (2012). Biochemical and metabolic profiles of *Trichoderma* strains isolated from common bean crops in the Brazilian Cerrado, and potential antagonism against *Sclerotinia sclerotiorum*. Fungal Biol..

[34] El Komy M.H., Saleh A.A., Eranthodi A., Molan Y.Y. (2015). Characterization of novel *Trichoderma asperellum* isolates to select effective biocontrol agents against tomato *Fusarium* wilt. Plant Pathol. J..

[35] Mwangi M.W., Muiru W.M., Narla R.D., Kimenju J.W., Kariuki G.M. (2019). Management of *Fusarium oxysporum* f. sp. *lycopersici* and root-knot nematode disease complex in tomato by use of antagonistic fungi, plant resistance and neem. Biocontrol Sci. Technol..

[36] Zhang F.L., Liu C., Wang Y., Dou K., Chen F., Pang L., Kong X., Shang C., Li Y. (2020). Biological characteristic and biocontrol mechanism of *Trichoderma harzianum* T-A66 against bitter gourd wilt caused by *Fusarium oxysporum*. J. Plant Pathol..

[37] Abo-Elyousr K.A.M., Abdel-Hafez S.I.I., Abdel-Rahim I.R. (2014). Isolation of *Trichoderma* and evaluation of their antagonistic potential against *Alternaria porri*. J. Phytopathol..

[38] Irene C.C., José L.T.C., Concepción O.G., Enrique M., Rosa H., Rafael M.J.D. (2016). *Trichoderma asperellum* is effective for biocontrol of *Verticillium* wilt in olive caused by the defoliating pathotype of *Verticillium dahlia*. Crop Prot..

[39] Swain H., Adak T., Mukherjee A.K., Mukherjee P.K., Bhattacharyya P., Behera S., Bagchi T.B., Patro R.S., Khandual A., Bag M.K. (2018). Novel *Trichoderma* strains isolated from tree barks as potential biocontrol agents and biofertilizers for direct seeded rice. Microbiol. Res..

[40] You J.Q., Zhang J., Wu M.D., Yang L., Chen W.D., Li G.Q. (2016). Multiple criteria-based screening of *Trichoderma* isolates for biological control of *Botrytis cinerea* on tomato. Biol. Control.

[41] Abbey J.A., Percival D., Abbey L., Asiedu S.K., Prithiviraj B., Schilder A. (2019). Biofungicides as alternative to synthetic fungicide control of grey mould (*Botrytis cinerea*)-prospects and challenges. Biocontrol Sci. Technol..

[42] Erazo J.G., Palacios S.A., Pastor N., Giordano F.D., Rovera M., Reynoso M.M., Venisse J.S., Torres A.M. (2021). Biocontrol mechanisms of *Trichoderma harzianum* ITEM 3636 against peanut brown root rot caused by *Fusarium solani* RC 386. Biol. Control.

[43] Yánez-Mendizábal V., Zeriouh H., Viñas I., Torres R., Usall J., de Vicente A., Pérez-García A., Teixidó N. (2012). Biological control of peach brown rot (*Monilinia* spp.) by *Bacillus subtilis* CPA-8 is based on production of fengycin-like lipopeptides. Eur. J. Plant Pathol..

[44] De Cal A., Larena I., Linan M., Torres R., Lamarca N., Usall J.M., Domenichini P., Bellini A., de Eribe X.O., Melgarejo P. (2009). Population dynamics of *Epicoccum nigrum*, a biocontrol agent against brown rot in stone fruits. J. Appl. Microbiol..

[45] Guijarro B., Melgarejo P., De Cal A. (2008). Influence of additives on adhesion of *Penicillium frequentans* conidia to peach fruit surfaces and relationship to the biocontrol of brown rot caused by *Monilinia laxa*. Int. J. Food Microbiol..

[46] Zhang D.P., Lu C.G., Zhang T.T., Spadaro D., Liu D.W., Liu W.C. (2014). *Candida pruni* sp. nov. is a new yeast species with antagonistic potential against brown rot of peaches. Arch. Microbiol..

[47] May-De Mio L.L., Negri G., Michailides T.J. (2014). Effect of *Trichothecium roseum*, lime sulphur and phosphites to control blossom blight and brown rot on peach. Can. J. Plant Pathol..

[48] Keske C., Amorimb L., May-De Mio L.L. (2011). Peach brown rot incidence related to pathogen infection at different stages of fruit development in an organic peach production system. Crop Prot..

[49] Lane C. (2002). A synoptic key for differentiation of *Monilinia fructicola*, *M. fructigena* and *M. laxa*, based on examination of cultural characters. EPPO Bull..

[50] Luo C.X., Hanamura H., Sezaki H., Kusaba M., Yaegashi H. (2002). Relationship between avirulence genes of the same family in rice blast fungus *Magnaporthe grisea*. J. Gen. Plant Pathol..

[51] Etebarian H.R., Sholberg P.L., Eastwell K.C., Sayler R.J. (2005). Biological control of apple blue mold with *Pseudomonas fluorescens*. Can. J. Microbiol..

[52] Zhang S.W., Xu B.L., Zhang J.H., Gan Y.T. (2018). Identification of the antifungal activity of *Trichoderma longibrachiatum* T6 and assessment of bioactive substances in controlling phytopathgens. Pestic. Biochem. Phys..

[53] Zhang S.W., Xiang D., Li T., Xu B.L. (2021). First report of brown rot of nectarine caused by *Monilia yunnanensis* in Tibet. Plant Dis..

[54] Zhao Y.Z., Wang D., Liu Z.H. (2013). First report of brown rot on *Crataegus pinnatifida* var. major caused by *Monilia yunnanensis* in China. Plant Dis..

[55] Yin L.F., Chen S.N., Chen G.K., Schnabel G., Du S.F., Chen C., Li G.Q., Luo C.X. (2015). Identification and characterization of three *Monilinia* species from plum in China. Plant Dis..

[56] Yin L.F., Cai M.L., Du S.F., Luo C.X. (2017). Identification of two *Monilia* species from apricot in China. J. Integr. Agr..

[57] Fu W.H., Tian G.R., Pei Q.H., Ge X.Z., Tian P.F. (2017). Evaluation of berberine as a natural compound to inhibit peach brown rot pathogen *Monilinia fructicola*. Crop Prot..

